# Assessment of Fabry variant (IVS4+919G>A) Cardiomyopathy in Taiwanese: Emphasis on T1 mapping

**DOI:** 10.1186/1532-429X-18-S1-P294

**Published:** 2016-01-27

**Authors:** Yu-pin Chang, Jyh-wen Chai, Yun-ching Fu, Yi-Ying Wu, Ying-xiang Liao, John Wang, Clayton Chi-Chang Chen

**Affiliations:** 1 Radiology, Taichung Veterans General Hospital, Taichung, Taiwan; 2 Pediatrics, Taichung Veterans General Hospital, Taichung, Taiwan; 3 Pathology, Taichung Veterans General Hospital, Taichung, Taiwan

## Background

Fabry disease is a rare X-linked disorder characterized by deficiency ofα-galactosidase A, leading to progressive accumulation of glycosphingolipid in various organs, including the heart. Several studies have pointed out the unique pre-contrast T1 value character of classic Fabry cardiomyopathy, which is lower than the normal myocardium. In Taiwan, several recent studies pointed out a surprisingly high incidence of a later onset Fabry mutation (IVS4+919G>A). There is a lack of information about the T1 value of this Fabry variant cardiomyopathy in the literature. We aim to present the T1 value of this subtype of Fabry disease on cardiac MR.

## Methods

As referred from pediatric cardiologist, we collected cases with Fabry mutation (IVS4+919G>A), proved via gene analysis from blood sampling and endomyocardial biopsy. They underwent MR study from March 2015 to July 2015. We recorded the location of delayed myocardial enhancement according to AHA 16-segment model and measured the thickest segment of left ventricular (LV) in short axis view at end diastole. We used conventional look-locker sequence (pre- and post contrast) and the Circle CVi42 software to calculate T1 value of target myocardium. Another 16 normal volunteers (with age and gender matched) were collected and they underwent non-contrast cardiac MR with T1 map evaluation. The myocardial pre-contrast T1 values were compared by t test as follows: Fabry cases vs. control group, Fabry cases with left ventricular hypertrophy (LVH, at least two segments ≥ 13 mm in thickness at end diastole) vs. Fabry cases without LVH. The pre-contrast T1 values are compared by paired t test as follows: whole LV myocardium vs. interventricular setum vs. LV inferio-lateral wall, enhanced myocardium vs. non-enhanced myocardium.

## Results

A total of 16 patients (11 males and 5 females, age: 58.1 ± 9.6) were enrolled in this study and the basic characters of LV were as follows: ejection fraction of LV=64.0 ± 10.5%, LV stroke volum=103.2 ± 28.3 ml, LV mass=167.4 ± 68.3 gm, LV mass index=94.5 ± 37.8 gm/m^2^. The T1 value of LV myocardium was significantly lower in Fabry variant cases than the normal control (759.1 ± 45.2 ms vs. 1026.6 ± 42.0 ms, *P* < 0.001). No significant difference of T1 value of LV myocardium was noted between Fabry variant cases with and without LVH (800 ± 55.1 ms vs. 667.5 ± 69.3 ms, *P*=0.181). The T1 value of whole LV myocardium and inferiolateral wall of fabry cases were both higher than that of interventricular septum of fabry cases (759.1 ± 45.2 ms vs. 168.4 ± 42.1 ms, 769.1 ± 44.0 ms vs. 168.4 ± 42.1 ms, both *P* < 0.001). In fabry cases with delayed enhancement of LV, The enhancing myocardium demonstrated higher T1 value than non-enhancing myocardium (853.2 ± 21.5 ms vs. 803.2 ± 21.9 ms, *P*=0.011).

## Conclusions

In our study, Fabry variant cases demonstrated lower pre-contrast T1 value of LV myocardium comparing with normal control cases. No significant difference of pre-contrast T1 value was noted between fabry variant cases with and without LVH.

**Figure 1:**
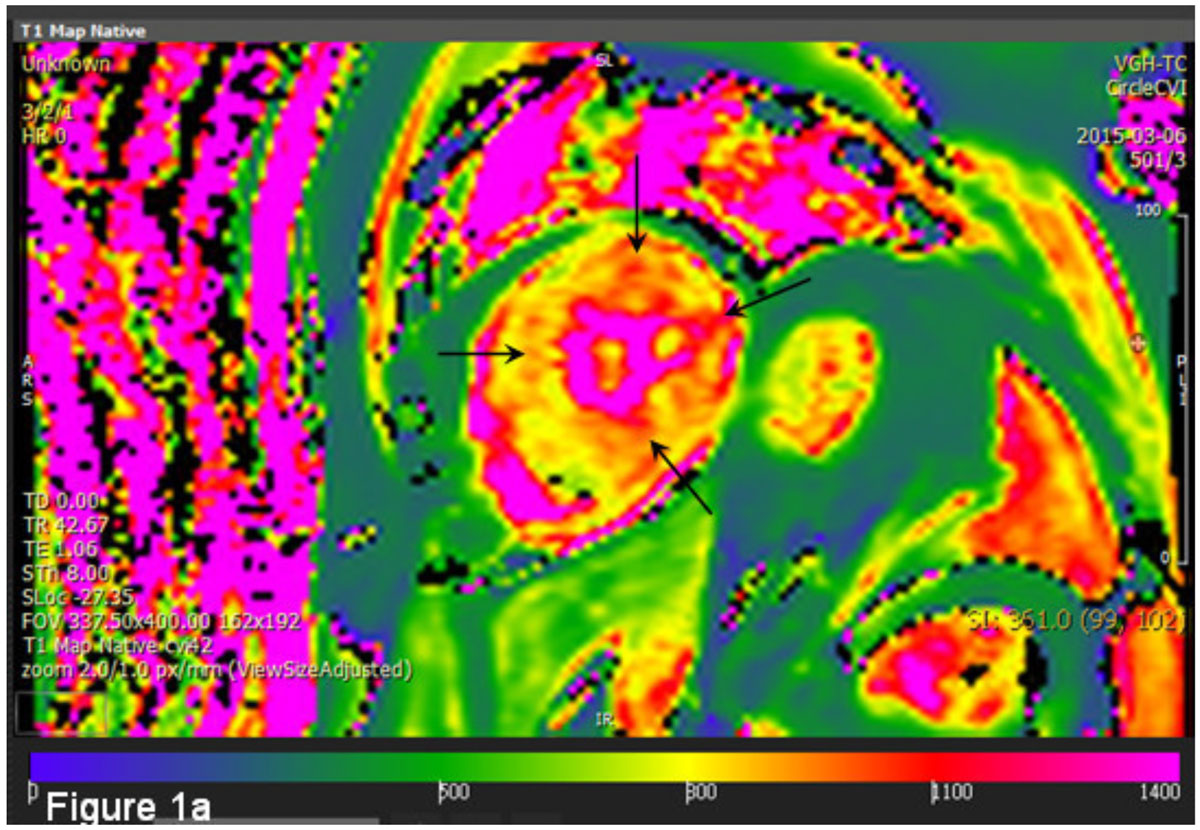
**A 63y/o male with fabry variant cardiomyopathy**. The T1 map generated by pre-contrast look-locker revealed generalized mildly low T1 value of LV myocardium (Average=875.2 ms). Several focal high T1 areas and foci are noted at mid anterioseptal, anteriolateral, inferiolateral and inferior walls (black arrows in Figure 1a), which generally corresponds to areas with delayed enhancement (gray arrows in Figure 1b).

**Figure:**
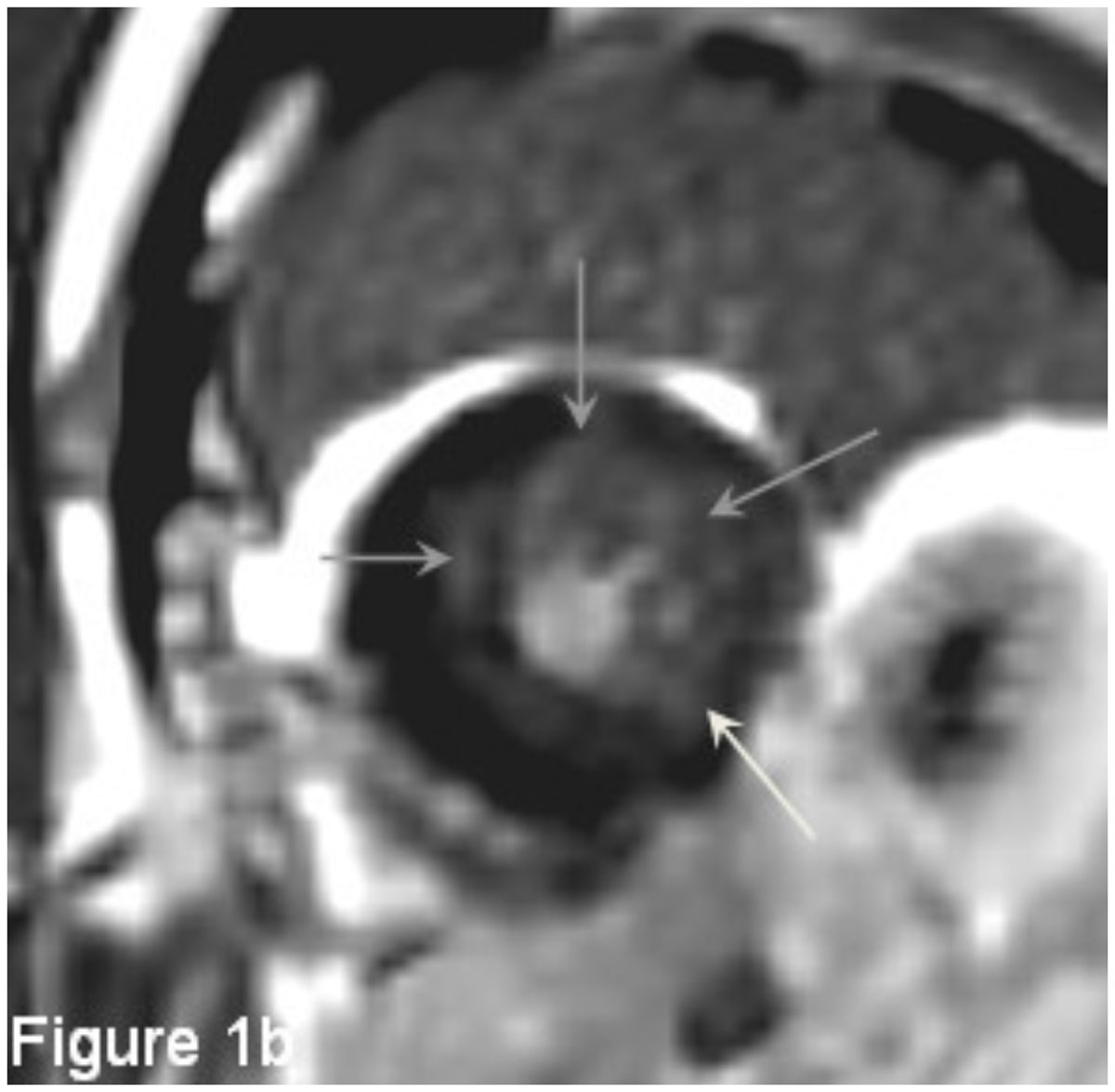
Figure 2

